# Recruitment, mortality and growth in semi‐arid conifer‐eucalypt forest: Small trees insure against fire and drought

**DOI:** 10.1111/jbi.14522

**Published:** 2022-12-13

**Authors:** Mathias Neumann, Chris S. Eastaugh, Mark A. Adams

**Affiliations:** ^1^ Faculty of Science, Engineering and Technology Swinburne University of Technology Melbourne Victoria Australia; ^2^ Institute of Silviculture University of Natural Resources and Life Sciences Vienna Austria; ^3^ Forestry Corporation New South Wales Dubbo New South Wales Australia

**Keywords:** Australia, *Callitris glaucophylla*, ecosystem restoration, *Eucalyptus crebra*, forest dieback, management, monitoring, pre‐European settlement, species mixture effects, wildfire impacts

## Abstract

**Aim:**

Well‐managed semi‐arid forests help offset global change by storing significant amounts of carbon above‐ and belowground and maintaining hydrological cycles. Larger trees have been the focus of many studies due to their carbon storage and habitat quality, yet recruitment and small trees are important components of ecosystem resilience and recovery. Here, we study the impacts of disturbances (including harvesting) on recruitment, mortality and growth for a mixed conifer‐broadleaf semi‐arid forest type using long‐term data.

**Location:**

Pilliga Forest in New South Wales, inland eastern Australia.

**Taxon:**

*Callitris‐Eucalyptus* forests.

**Methods:**

We used data from permanent sample plots (PSPs) spanning 55 years, calculated stand structure, gains and losses and determined reasons for tree death (harvesting, fire, wind, drought and other effects). We extracted climate and fire data for the PSP locations using spatial analysis.

**Results:**

Stocking of studied forests remained stable (modest increase in basal area and stem density), despite harvesting and wildfires over 6 decades. Compared to stands in the 1940s and prior to European settlement, current forests are composed of more trees per unit area, and these trees have smaller diameters. Recruitment and sustained presence of small trees have buffered impacts of recurring drought, fire and harvesting. Fires are a common feature of the studied ecosystems and fire impacts have increased in the past 20 years, especially in unmanaged stands, where fires have reduced tree carbon by >50%.

**Main conclusions:**

Recruitment and growth of small trees are critical to offset carbon losses due to fire, drought and harvesting. All size classes have important ecological values in semi‐arid forests and must be included in long‐term monitoring programmes. Long‐term data offer unique insights into combined effects of climate change, management and disturbances, especially for fire‐prone ecosystems, where small trees are often susceptible to fire.

## 1 INTRODUCTION

Aridity, that is, the degree of climatic dryness, is frequently considered as a key driver in biogeographical studies as it has been shown to correlate well with changes in vegetation types and distributions of individual species scales from local to continental. A commonly used aridity measure is the Aridity Index (AI, ratio of annual rainfall to annual potential evapotranspiration). About 18% of all terrestrial land surfaces have AI in the range from 0.2 to 0.5. These areas are classified as having ‘semi‐arid’ vegetation, that, collectively comprises one of the largest classes of vegetation globally (Cherlet et al., 2018; Middleton & Thomas, 1992). In these challenging climates, common vegetation types include slow‐growing forests and woodlands. Examples include Pinyon‐Juniper woodlands in North America, South American Caatinga, Miombo woodlands in Africa, deciduous dipterocarp forests in Asia and Mediterranean Pine forests in Southern Europe (Chidumayo, 2019; Grier et al., 1992; Guller et al., 2012; Nguyen & Baker, 2016; Paloschi et al., 2021). In the Australian inland, conifer‐eucalypt forests cover large areas and have considerable ecological, historical and commercial significance. While individual trees in these ecosystems are usually smaller in stature than their counterparts in coastal and moist forests, maximum tree diameters in semi‐arid forests often exceeds 60–80 cm (Ngugi et al., 2015; Ryan et al., 2011). Larger trees are frequently described as main drivers of overall forest growth and net primary production (Prior & Bowman, 2014; Stephenson et al., 2014); a feature often underscored by the process of self‐thinning (mortality of smaller trees due to competition).

*Callitris* spp. (‘Cypress pine’) are widespread, slow‐growing conifers endemic to Australia and New Caledonia, covering around two million hectares (~2% of the total forest area) in Australia (Montreal Process Implementation Group for Australia and National Forest Inventory Steering Committee, 2018), making this genus a significant contributor to the extent of dry climate forests worldwide. *Callitris* spp. occur in woodlands encompassing annual rainfalls from <300 mm to more than 1000 mm and are subject to frequent droughts (Brodribb et al., 2013; Prior et al., 2012). *Callitris* spp. are mostly obligate seeders, capable of surviving low‐intensity fires but sensitive to intense fires. The most common *Callitris* spp. are *Callitris glaucophylla* or White Cypress pine (J.Thomps. & L.A.S.Johnson), *Callitris intratropica* or Blue Cypress pine (R.T.Baker & H.G.Sm.) and *Callitris endlicherii* or Black Cypress pine (Parl., F.Muell.). Taxonomic uncertainty exist regarding differentiation of *C. glaucophylla* and *C. intratropica* (Farjon, 2005). Previous studies have documented aspects of the growth, and fire‐ and drought‐sensitivity of the genus (Cohn et al., 2011; Denham et al., 2016; Prior et al., 2012). *Callitris* spp. often regenerate and grow under canopies, which suggests that their growth is limited by factors other than light, albeit that light is seldom limiting in inland Australian forests and woodlands owing to open and sparse canopies. Their slow growth, limited accumulation of litter and high bulk density of litter layer on the forest floor helps reduce their risk of burning (Bowman et al., 2018; Clayton‐Greene & Ashton, 1990; Hart, 1995; Trauernicht et al., 2012). Mixed *Callitris‐Eucalyptus* forests have been important sources of timber since the arrival of European settlers (Ross et al., 2012; Thompson & Eldridge, 2005).

Through changing land‐use and forest management, humans have altered the extent of *Callitris‐Eucalyptus* forest in Australia considerably and there is concern about the future and stability of these ecosystems and appropriate management (e.g. Lunt et al., 2006; Ngugi et al., 2013; Trauernicht et al., 2016). Improved understanding of ecosystem resilience is needed to interpret historic and future stability of biogeographic zones (Colloff & Baldwin, 2010; Van Meerbeek et al., 2021; Werneck et al., 2012).

The largest remaining contiguous *Callitris‐Eucalyptus* forest in Australia is the ‘Pilliga scrub’ in New South Wales (400,000 ha). Other large examples are found in Queensland (Ngugi et al., 2013). Prior to European settlement, analysis suggests the Pilliga scrub and other mixed *Callitris*‐*Eucalyptus* forests were mostly open woodlands (Lunt et al., 2006; Rolls, 1999), with their structure presumably maintained by the burning practices of indigenous Australians, as evident for Northern Australia (Russell‐Smith et al., 2013). Typically, management for timber production of these forests can be described as ‘single‐tree’ and ‘group selection’, whereby more highly valued *Callitris* spp. are selectively harvested, often leaving the eucalypts (Lacey, 1973). Other human‐led activities affecting these forests are livestock grazing, mineral exploration and extraction of natural gas.

Field surveys were conducted in the Pilliga by the New South Wales Forestry Commission in the 1920s/30s and the 1940s/50s (Forestry Commission of New South Wales, n.d., 1946) and since 1964 using a permanent plot system (details in the Method section). Whipp et al. (2012) analysed a subset of available field data and found that both basal area and stem density increased over 60 years, predominantly by *Callitris* spp. Anecdotal evidence discussed by Rolls (1999) points in the same direction, that the Pilliga scrub has experienced profound changes in stand structure and was composed of fewer but larger trees in the past. Attempts (e.g. Lunt et al., 2006; Whipp et al., 2012) to understand the growth ecology of mixed *Callitris*‐*Eucalyptus* forests and woodlands have been restricted by availability of quantitative data. Two intense wildfires, in 2006/07 and 2017/2018 (Department of Planning Industry and Environment, 2020; Whipp et al., 2012) swept through large proportions of the Pilliga scrub, but their effects on growth and structure from tree to stand level have not been reported. Understanding slow gradual changes of vegetation, such as observed global vegetation greening (Donohue et al., 2009; Yan et al., 2016) also requires long‐term field data.

Previous work has demonstrated that large trees have faster increment and more carbon accumulation than their smaller counterparts (Prior & Bowman, 2014; Stephenson et al., 2014) leading us to hypothesize that aboveground growth of semi‐arid *Callitris*‐*Eucalyptus* forests would be dominated by growth of the largest trees. We further hypothesize that mortality via self‐thinning (and not climate and droughts) would drive changes in stand structure determining ecosystem stability in semi‐arid *Callitris*‐*Eucalyptus* forests, due to reported abilitiy of the genus *Callitris* to endure high levels of stress and competition, in the absence of fire (Horne, 1990; Trauernicht et al., 2012). To test our hypotheses, we linked inventory data covering 55 years with climate data and fire records as external environmental drivers, and complemented our data with stand assessments dating back to pre‐European settlement.

## 2 MATERIALS AND METHODS

### 2.1 Study area

**FIGURE 1:**
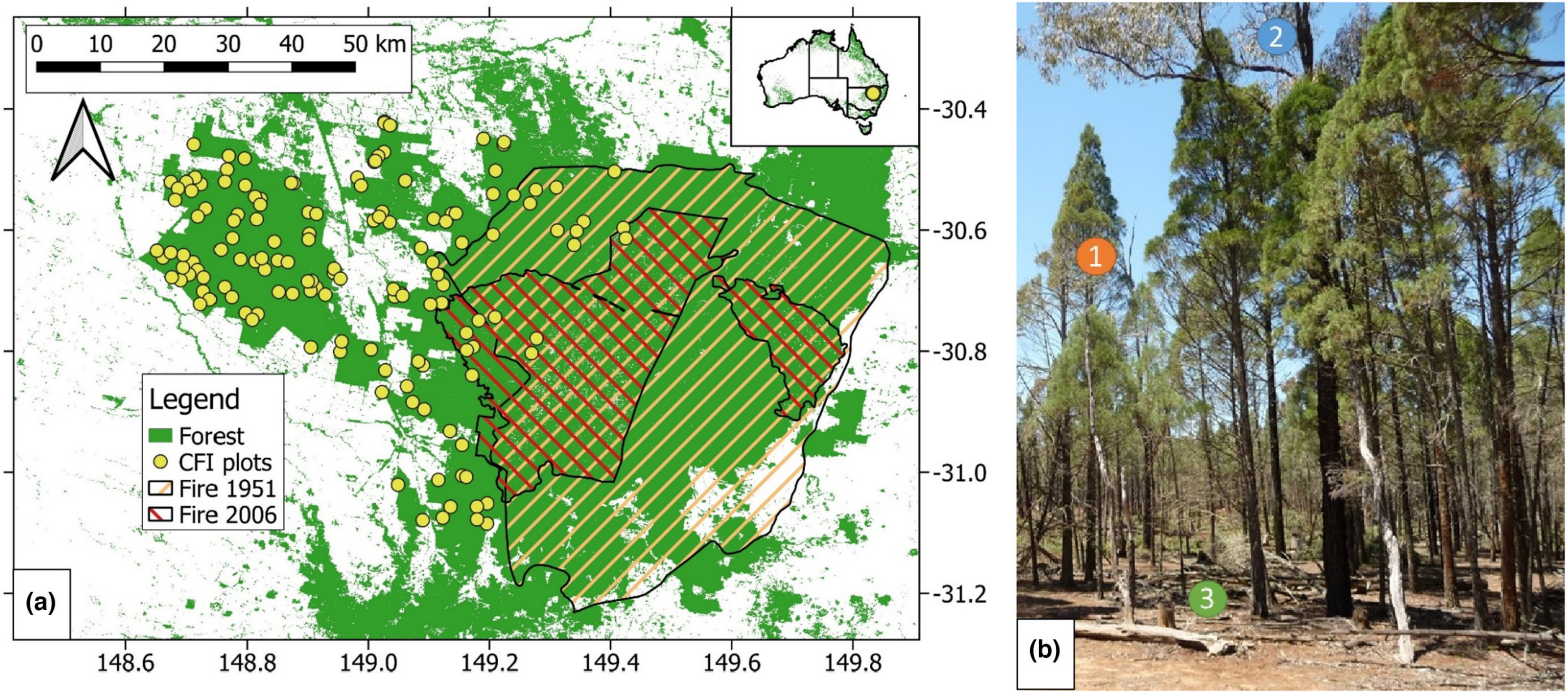
Location of Continuous Forest Inventory (CFI) plots, forest cover and extent of wildfires in 1951 and 2006 (Department of Planning Industry and Environment, 2020) in panel (a). The extent of the 2017 fire (boundary not shown) is mostly within the perimeter of the 2006 fire. Map projection is WGS84 (EPSG:4326). Panel (b) shows typical stand structure of a Callitris‐Eucalyptus forest. 1 (orange circles) shows *Callitris glaucophylla*, often underneath the canopies of *Eucalyptus crebra* (2, blue). This site has been recently thinned, as evident from slash and stumps (3, green)

Figure 1b shows the typical stand structure of mixed *Callitris‐Eucalyptus* forests, that dominate the Pilliga (for an overview, see Figure 1a). The average stand height is 15–20 m with Eucalypts dominating the overstory and *Callitris* spp. the lower strata. Depending on management history, species proportions vary considerably with managed stands having greater proportions of *Callitris* contributing to measured stem density and basal area. Common species in the Pilliga include *C. glaucophylla, C. endlicherii*, *Eucalyptus crebra* F. Muell. (Narrow‐leaved ironbark), *Eucalyptus pilligaensis* Maiden (Pilliga box), *Eucalyptus populnea* F. Muell. (Poplar box), *Eucalyptus blakelyi* Maiden (Blakely's red gum) and *Allocasuarina luehmannii* (Aiton) L. A. S. Johnson (Buloke). Canopy cover of unburnt stands rarely exceed 60% and crown length is usually between one‐third and half of tree height (Figure 1b). Fire causes pronounced changes in stand structure, owing to the sensitivity of *Callitris* (killed by intense fire; Trauernicht et al., 2012). Larger *Callitris* can survive fires of lesser intensity due to moderately thick bark (Lawes et al., 2011). Nonetheless, resprouting eucalypts generally dominate post‐fire. Slow growth and recruitment of fire‐sensitive species, including *Allocasuarina* spp., eventually moderate the dominance of eucalypts.

*Callitris‐Eucalyptus* forests commonly regenerate in ‘pulses’ or ‘waves’ (Horne, 1990; Horne & Robinson, 1987) that arise from wet years and fires following periods of drought, leading to dense stands of seedlings that pose challenges for management. Such stands can remain in this stage if undisturbed or unthinned and are sometimes referred to as ‘locked stands’ (Baur, 1990; Horne, 1990). Tree age then becomes an ineffective proxy for growth, as two trees of same age can have widely different dimensions. This phenomenon is well illustrated in the Strahorn State Forest, where, depending on stocking, stands aged 40 years could have (a) a mean diameter of ~3 cm and a mean height of ~2 m or (b) a mean diameter of ~15 cm and height of ~10 m (Baur, 1990). *Callitris* spp. are frequently found alongside faster‐growing *Eucalyptus* spp. that are more often the subject of research. Diameter increments for *Callitris* spp. are also often at the lower end of the range of those for associated *Eucalyptus* spp. (Ngugi et al., 2015). Stands of *Callitris* are thus notably and frequently characterised by many small trees (e.g. 90% <10 cm) usually in mixtures with larger eucalypts (Whipp et al., 2012).

### 2.2 Data

The Pilliga has an unusually good history of mensuration. We used data from the Pilliga Continuous Forest Inventory (CFI) which began in 1964 with the establishment of 144 Permanent Sample Plots (circular PSP, radius = 17.94 m, 0.1011 ha, Turner, 1968). One hundred and thirty‐six of these PSP have been assessed five times (1964, 1972/73, 1984/85, 2000/01 and 2019). In each assessment, all trees >10 cm diameter at breast height (DBH) were identified (species) and then measured for DBH and height and status (and other tree properties) and permanently marked. Status includes ‘new’ trees reaching the diameter threshold of 10 cm (recruitment) and comments as to why a tree died.

We aggregated tree death information into four groups (harvesting, wind, fire, other). If a dead tree was broken or uprooted, we assumed it was killed as a result of wind. If a dead tree was charred and/or the crown was scorched, we assumed it was killed by fire. Unburnt standing dead trees were assumed to have been killed by drought (or related conditions). There is scant and only dated information available on the role of fungal and insect diseases for tree mortality (Lacey, 1973). Tree death information becomes more uncertain in more recent years owing to greater periods between measurements (approaching two decades). While harvesting effects are mostly obvious (remaining cut stumps), we expected greater uncertainty in attributing death to fire or drought.

Information prior to 1964 was extracted from literature, based on transect measurements and reconstructions using stumps (Lunt et al., 2006; Whipp et al., 2012) and include different levels of uncertainty, compared to the consistent method of the CFI.

### 2.3 Stand structure, gains and losses

We focused on diameter, since tree age is not suitable for evaluating growth response (Baur, 1990) and tree height has not been routinely measured since 1964 with comparable accuracy. For each PSP, we extracted data on stem density (ha^−1^) and basal area BA (m^2^ ha^−1^, Equation 1) using tree diameter at breast height (DBH, cm) and PSP size (S) of 1011 m^2^.

(1) $$\mathrm{BA}=10,000/S\cdot \sum \left({\mathrm{DBH}}^{2}\cdot \pi /40000\right).$$

For the four periods with repeated measurements (1964–73, 1972–85, 1984–01, 2000–19), we calculated gains in basal area by surviving trees (basal area increment, BAI) (m^2^ ha^−1^ year^−1^) and stem density (stems ha^−1^ year^−1^) correcting for measurement period as follows:

(2) $$\mathrm{BAI}=\left(\text{BA2}-\mathrm{BA}1\right)/\text{period in years}.$$

For recruitment—the second important gain in basal area at the plot scale—we used Equation 2 with BA1 = 0. For mortality and harvesting (losses of basal area at the plot scale), BA2 = 0. Basal area and stem density were preferred to stem volume owing to a lack of suitable volume functions. For trees <10 cm DBH, we used data in the form of counts made in 1972/73, 1984/1985 and 2019 and calculated their stem density (ha^−1^). For 5–10 cm trees, we used measurements made on a sub‐sample of 29 PSP between 1980s and 2019.

### 2.4 Climate data and fire history

We sought to reconcile observed changes in stand structure, growth, recruitment and mortality with ‘SILO’ gridded climate data (Jeffrey et al., 2001). SILO is a regional climate product for Australia, maintained by the Australian Bureau of Meteorology and the Queensland Government and currently available over https://www.longpaddock.qld.gov.au/silo/. The spatial resolution is 0.05° by 0.05°. We extracted annual average temperature, annual precipitation sum and potential evapotranspiration using the plot locations to check for patterns over time. We then calculated periodic averages for the measurement periods as input for mixed models. Government records of wildfires affecting the PSPs were available for 1951/52 (about 382,000 ha, prior CFI measurements), 1982/83 (about 110,000 ha), 1997/98 (about 137,000 ha), 2006/07 (about 148,000 ha) and 2017/2018 (about 60,000 ha) (Department of Planning Industry and Environment, 2020; Whipp et al., 2012). Smaller, more localized fires in the eastern Pilliga, that did not affect the PSPs, occurred in 1957/58 (65,000 ha), 1965/66 (65,000 ha), 1974/75 (43,000 ha), 1976/77 (22,000 ha), 1977/78 (25,000 ha), 1978/79 (18,000 ha) and 2002/03 (22,000 ha) (Department of Planning Industry and Environment, 2020). The eastern part of the forest receives more rainfall and experiences more fires than the western part, which remained mostly unburnt since beginning of records (Figure 1a, Figure S1). In semi‐arid ecosystems, fire activity and rainfall are often linked through increased fuel loads (e.g. Avitabile et al., 2013; Murphy, Bradstock, et al., 2013; Nolan et al., 2019; Turner et al., 2021).

We employed mixed models to help identify relations among gains and losses, stand structure and climate and the effect of observation period. After testing several potential covariates and their combination, we used as fixed effects: basal area (proxy for inter‐tree competition and biomass); quadratic mean diameter (proxy for tree size and mean stand age); precipitation (proxy for water availability). Observation period was the random effect.

**FIGURE 2:**
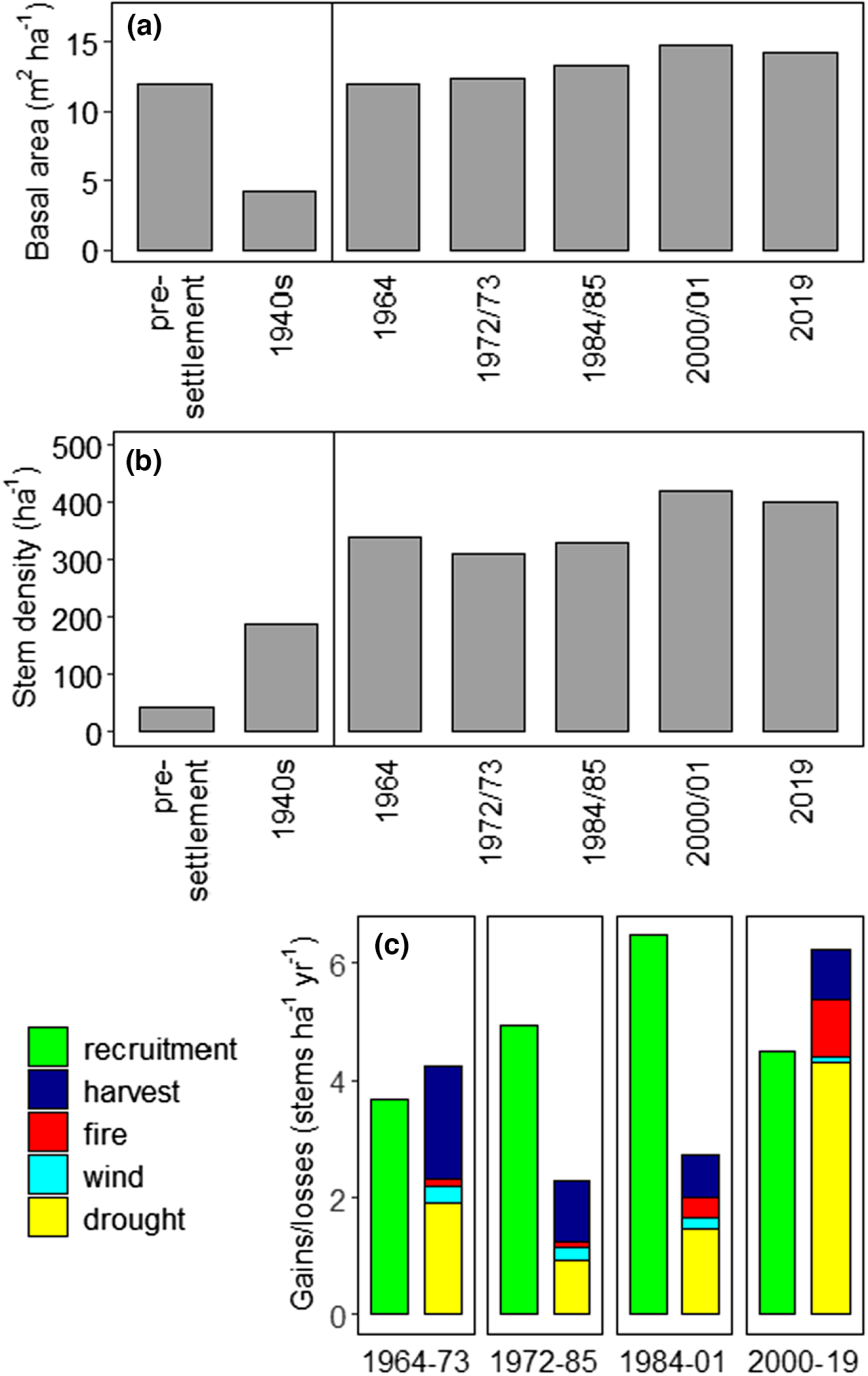
Basal area (a) and stem density (b) from pre‐European settlement until present for the Pilliga forest (stems >10 cm DBH, including all woody tree species). Pre‐European settlement values in (a) and (b) are based on Lunt et al. (2006) and Rolls (1999), while 1940s (1946–1951) data are derived from Whipp et al. (2012). From 1964 onwards, data are derived from the CFI. Annual gains (recruitment) and losses (harvesting/mortality) (c) explain changes in stem density between the five CFI measurements. Recruitment (shown in green) represents new additions to >10 cm diameter class while losses due to harvesting/mortality are grouped into four classes.

We used R statistical software (R Development Core Team, 2021) for all analysis and for figure preparation. Figure 2 was prepared using open‐source geographic information system, QGIS.

## 3 RESULTS

### 3.1 Forest structure of semi‐arid mixed *Callitris*‐*Eucalyptus* forests

While changes in basal area in the Pilliga were modest across the period for which data are available, stem density has mostly increased since European settlement (Figure 2). Stem density, as well as basal area, peaked in 2000/01 (Figure 2). Since 1964, there has been only modest turnover (losses due to mortality, gains due to recruitment) in tree populations, averaging 1.2% per year, with losses exceeding gains between 1964 and 1973 and again between 2000 and 2019 (Figure 2c).

Estimates of basal area for Pilliga forests prior to European settlement, and for mixed *Callitris*‐*Eucalyptus* forests more generally, are mostly around 12 m^2^ ha^−1^ (Lunt et al., 2006; Rolls, 1999), similar to that recorded for 1964–2019 (12.0–15.1 m^2^ ha^−1^, Figure 2a). Pre‐European settlement (mid‐1800s) density was far less (44 ha^−1^) than recorded in the 20th and 21st centuries (200–400 ha^−1^, see Figure 2b). Basal area and stem density changed little between 1964 and 2001 (Figure 2a,b). Variation in basal area was smaller than for stem density (see also Table S1). Species composition has also changed little since 1964 (Figures S2 and S3). Gains in stem density prior to 2001 were caused by large numbers of trees reaching a diameter of 10 cm, with small losses to mortality (Figure 2c). Losses in basal area and stem density due to harvesting were smaller than losses due to drought and fire, except between 1964 and 1973.

**FIGURE 3:**
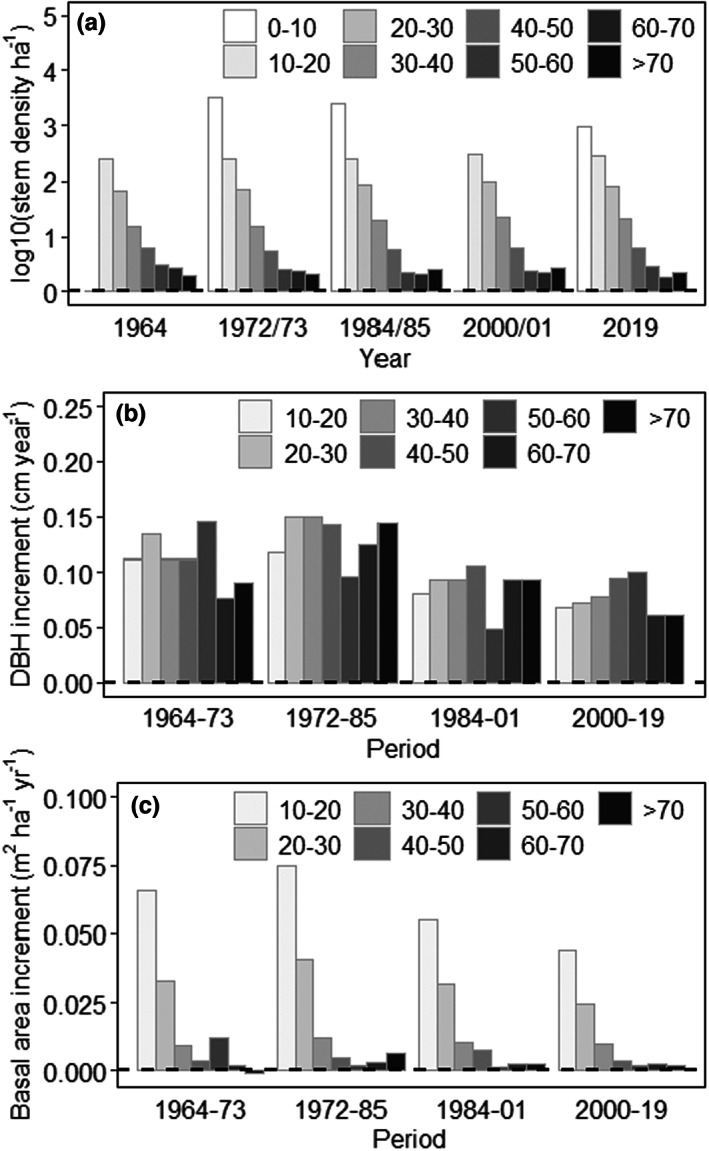
Variation in stem density (scaled using decadic logarithm; a), diameter increment (b) and basal area increment (c) with diameter class (indicated by grey scales). Smallest trees (0–10 cm) were assessed in 1972/73, 1984/85 and 2019. All data shown are medians derived from CFI (see text) and include all woody tree species.

Grouping by diameter reveals the strong influence of the smallest trees on overall stem density (Figure 3a). That contribution changed little over 50 years. Diameter increments of surviving trees were mostly about 0.1 cm year^−1^ (Figure 3b) irrespective of tree size. Larger increases in diameter increment for the 50–60 cm class between 1964 and 1973 and the 70+ cm class between 1972 and 1985 (Figure 3b) are reflected in larger basal area increments (BAI) for those periods (Figure 3c). Otherwise, BAI is dominated by stem density rather than by diameter increment. Contributions of smaller trees to overall BAI were remarkably constant (Figures 3c, Figure S4). Trees >10 and <20 cm diameter contributed more than 50% over the 50‐year period, with another 25% contributed by trees 20–30 cm diameter.

Assessments of major causes of tree death (= standing dead, fallen or removed trees) highlight the role of drought (Figures 2c, Figure S5), with little influence of wind or harvesting. Fire had a significant influence (~15%) in the latest measurement period (2000–2019). Lumped with drought are pathogenic insect activity, fungal disease and suppression (competition from other trees). Species identity had little influence on mortality apart from harvest impacts (Figures S2, S3, S5).

Available data for the smallest trees (<10 cm diameter; three assessments; Figure 3) reveal their greater density (ha^−1^) than all other sizes combined (Table S3). Diameter increments, by contrast, were remarkably consistent across all classes, including the 5–10 cm class (Figure 3b, Table S3). Considering the already substantial contribution of trees 10–20 cm (Figure 3c), contributions of small trees to overall BAI are even greater than the 50% + shown in Figure S4.

### 3.2 Reasons for changes in forest structure

**FIGURE 4:**
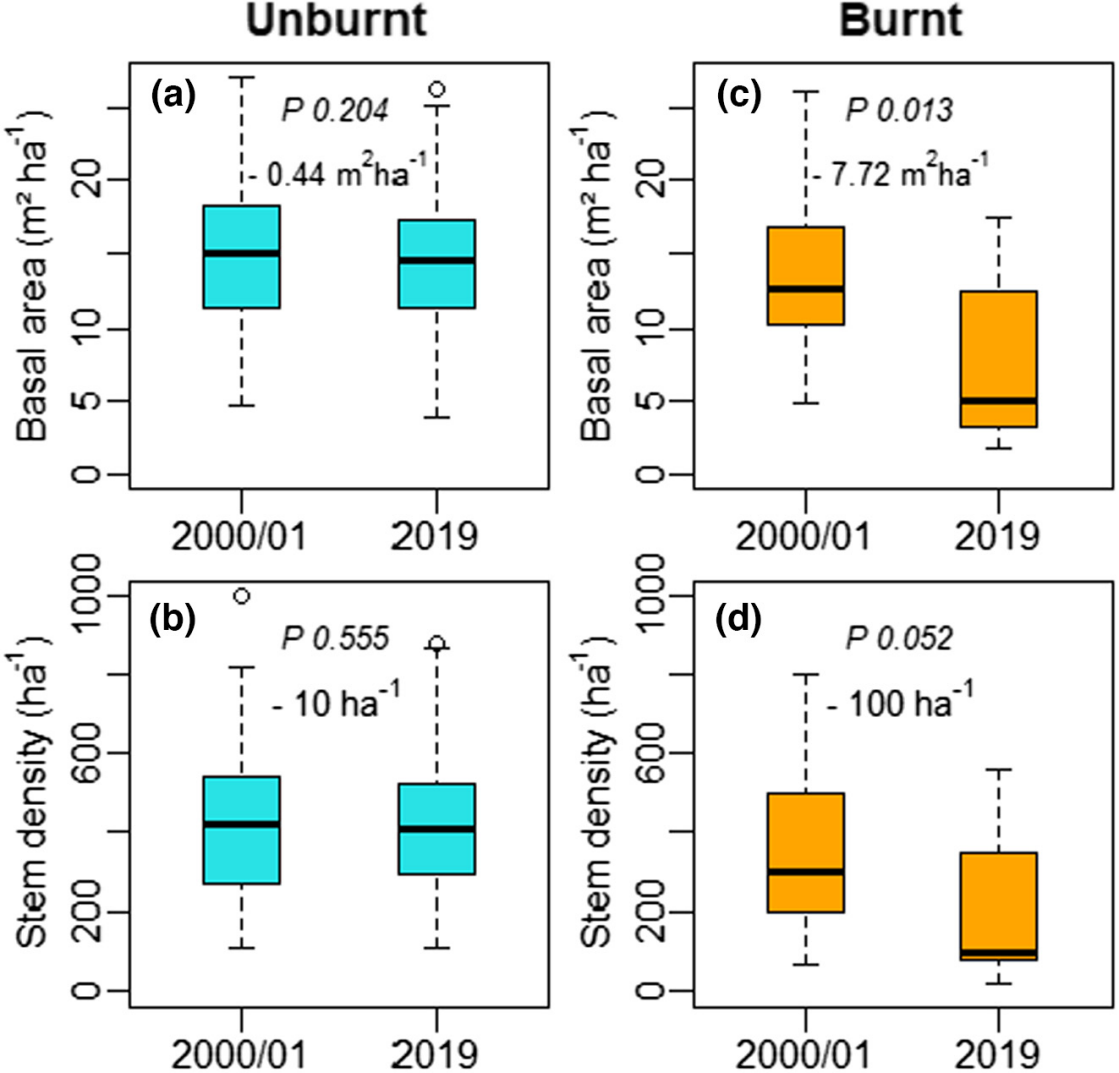
Effects of fire on basal area and stem density between 2000 and 2019. Unburnt PSP (*n* = 123, panels a and b) are compared to PSP burnt in 2006 and/or 2017 (*n* = 13, panels c and d). *P* values are derived from pairwise analysis of covariance and we show the difference in median between the 2000/01 and 2019 measurements. The box represents the median and the 25th and 75th percentiles, the whiskers extend to 1.5 of the interquartile range. Values outside this range indicated as circles.

Government records show that 13 PSP were affected by wildfires in 2006/07 and 2017 (Figure 1a). Fire reduced basal area from 13.3 to 7.7 m^2^ ha^−1^and stem density from 369 to 211 ha^−1^ compared to unburnt PSP (Figure 4). Unburnt PSP showed little variation despite significant droughts in intervening years (Figure S6).

Nevertheless, drought was the most common cause of tree death (Figures 2c, Figure S5). Standing dead trees were common after 2000. In two of the last 20 years, annual rainfall was less than the 5th percentile (342 mm year^−1^, Figure S6). Two large fires in same period must also be considered in light of above‐average temperatures, below‐average rainfall and the least aridity index (AI) on record (+0.24°C, −44 mm year^−1^, AI 0.30, Table S4). Over the 50 years of CFI measurements, basal area showed significant positive relationships with BAI and losses in basal area (*p* < 0.001), while correlation with recruitment was negative (Table S5). There was no regeneration pulse or wave during the period of CFI. Quadratic mean diameter was significantly and negatively correlated with BAI, and with losses and recruitment in basal area. Precipitation was positively correlated with all three variables. In summary, BAI were greater in stands with large basal area, smaller mean tree diameter and when precipitation was greater than its long‐term mean.

**FIGURE 5:**
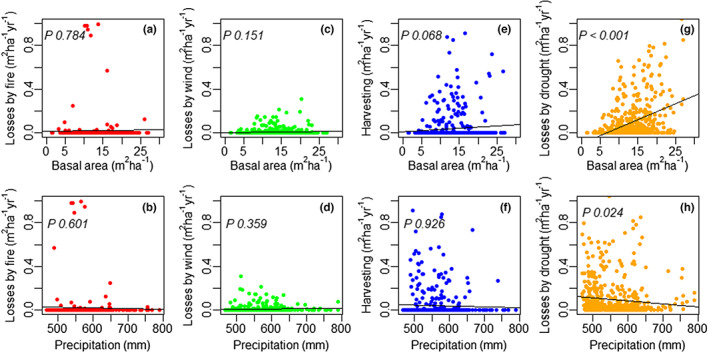
Relationships of losses of basal area due to fire (panels a, b), wind (panels c, d), harvesting (panels e, f) and drought (panels g, h) with basal area and precipitation (Jeffrey et al., 2001) respectively. Basal area (panels a, c, e, g) represents conditions at first measurement and precipitation (panels b, d, f, h) is the average for the remeasurement period between first and second measurement, used for deriving losses. We show results for all four remeasurement periods of the 136 plots (*n* = 548). Linear trend lines and their *p* values are shown for reference.

Losses of basal area due to drought were significantly positively related to basal area (*p* < 0.001, Figure 5, Table S5), while no other reason for mortality has significant correlation with basal area or precipitation. We note that losses by drought were more frequent when precipitation <600 mm year^−1^. The (positive) effect of basal area on BAI and on losses in basal area was greater for the period 2000–2019 than for 1984–2001 (Figure S7, Table S5).

In mixed models, random effects (observation period) explained around one‐third of the variation in BAI accounted for by fixed effects (basal area, quadratic diameter, precipitation). For recruitment and losses in basal area, random effects explain as much variation as fixed effects. Hence, climate and stand metrics only partly capture the dynamics of these semi‐arid forests. Stand structure has contrasting effects on carbon fluxes with greater basal area associated with greater BAI and losses, but lesser recruitment (Table S5). As a proxy for tree age, average diameter was negatively related to all gains and losses in basal area—stands composed of larger trees have reduced carbon fluxes.

## 4 DISCUSSION

### 4.1 Stability of semi‐arid forests assessed using long‐term data

Semi‐arid vegetation covers about 18% of the global land surface. Regionally, this vegetation has even greater importance. Western North America, Central Asia and Australia all have large areas of semi‐arid woodlands and forests (Cherlet et al., 2018) and numerous authors have drawn attention to the potential losses of carbon, habitat and local livelihoods that could stem from their degradation (e.g. Charro et al., 2017; Trumper et al., 2008; Werneck et al., 2012). Long‐term data revealed contrasting changes in carbon accumulation in semi‐arid Miombo woodlands, with gains in regrowth and losses in old‐growth stands, due to fire and harvesting (Chidumayo, 2019). Data for tropical forests also show globally significant, age‐related patterns in carbon sequestration (Hubau et al., 2020; Murphy, Bradford, Dalongeville, Ford, & Metcalfe, Murphy, Bradford, et al., 2013). Our study suggests that over six decades, semi‐arid mixed *Callitris‐Eucalyptus* forests in Australia showed considerable resilience in the face of changing climate, fire and harvesting. Carbon accumulated in aboveground biomass from 1940s until about year 2000. The data also reveal recent reductions in tree density and basal area on landscape scale.

Foresters, ecologists and historians agree that the age (and physical) structure of contemporary Pilliga forests results in large part from an intense regeneration pulse around 1880s (Rolls, 1999; Thompson & Eldridge, 2005; Whipp et al., 2012). Those historic effects were compounded by sequential effects of drought (World War II drought; 1937–45), years of increased rainfall (1946–1950), followed by the fire in 1951 (Figure S6). This created another recruitment ‘pulse’ (Whipp et al., 2012). Trees <20 cm diameter now represent the largest share of basal area, stem number, BAI and losses of basal area (Figure 3, Figure S4). Our results reject our hypothesis that it is the largest trees that drive overall forest growth (and net primary production) for these semi‐arid, mixed *Callitris‐Eucalyptus* forests. Instead, *Callitris* shows remarkably little self‐thinning. This feature is revealed in the largely constant stem density and small average diameter. This pattern has been maintained for over 50 years (Figure 3). Without further perturbation, notionally fire‐sensitive *Callitris* may eventually become more dominant than at present, displacing eucalypts (Clayton‐Greene & Ashton, 1990; Trauernicht et al., 2012). Harvesting at current rates has had minimal impact (Figure 2, Figure S1). In mixed semi‐arid forests in North America, a recent study using field data found higher mortality rates of broadleaved species compared to conifers (Assal et al., 2016). Their remote sensing analysis, however, suggested that the share of broadleaves has been increasing in the last two decades. Allthough drought and not fire was the main driver for changes in stand structure in the Assal et al. (2016) study, combining remotely sensed vegetation data with fire records will help further elucidate the observed changes in the Pilliga forests.

The studied forests are clearly semi‐arid (AI = 0.35, Cherlet et al., 2018) and characterized by large variability in annual precipitation (mean 565 mm, standard deviation 172 mm), which can fall to <250 mm in severe drought years (Jeffrey et al., 2001). On average, rainfall is <340 mm at least one year in 20 (Figure S6). While not exceptional for semi‐arid conifers globally (Lopez‐Lopez et al., 2013; Rau et al., 2009; Szejner et al., 2020), rainfall variation must be considered in light of average annual temperatures >18°C (Table S4) and an increase in fire frequency (Figure S1). Large fires (e.g. 1951/52, 2006/07 and 2017/18) mostly follow years of greater‐than‐average rainfall (Figure S6). Changes in year‐to‐year variation (switching from wet to dry conditions, see also Figure 5) can result in a build‐up of fine fuels and seem likely to be as important to fire frequency as long‐term reductions in rainfall. Increments were least across size classes in the 2000–2019 period (and mortality greatest, Figures 2 and 3), coinciding with smallest annual rainfalls and highest temperatures. Neither fire nor harvesting alone explain the greater losses in stem density after 2000 (Figure 2). Harvesting removed <1 stem ha^−1^ year^−1^ and only a small portion of the total area of the Pilliga forest burnt in any single year.

Analyses using remote sensing suggest that large areas of the globe have ‘greened up’ since the 1980s, presumably due to CO_2_ fertilization, climate warming and changes in water use efficiency, with likely implications for the global carbon balance (Adams et al., 2021; Donohue et al., 2009; Liu et al., 2021). While the present study cannot test this suggested change, our data support ‘vegetation thickening’ in terms of increasing basal area and stem density from 1964 until present (Figure 2). However, more frequent wildfires can counter this trend in the most recent decades. More limited inputs of atmospheric nitrogen to southern hemisphere ecosystems (Ackerman et al., 2019) bring into question direct translation of long‐term trends in semi‐arid forests in the northern hemisphere (e.g. Pinyon‐Juniper woodlands or dry deciduous dipterocarp forests) to forest and woodlands like those in the Pilliga.

### 4.2 Implications for carbon and management of semi‐arid forests

Forest management has been reported to be responsible for accumulating a carbon debt, increases in atmospheric CO_2_ and fire risk in temperate and boreal forests (e.g. Lindenmayer et al., 2009; Naudts et al., 2016). However, in mixed‐species, semi‐arid Pilliga forest, management has had a negligible effect for the past 50 years. Prior to this period, forest management focused on meeting economic demand for different species. For example, many of the larger eucalypts were either harvested or killed by ring‐barking and poisoning in the period between 1930 and 1960 (Baur, 1990; Whipp, 2009). In part, this strategy was designed to encourage growth of larger individuals of valuable *Callitris* spp. Available evidence suggests harvesting had reduced basal area to about one‐third of its pre‐European settlement condition by the 1940s (Figure 2). We urge caution when comparing 1940s data based on transects and 1964–2019 data based on permanent sample plots (Whipp et al., 2012). After the post‐war introduction of sustainable harvesting based on survey, basal area recovered to pre‐European settlement condition by 1964 (and then slowly increased until around 2000). Most recently, gains in basal area have stagnated likely due to the interactive effects of climate and fire (as outlined above). While we cannot exclude non‐drought causes, the weight of evidence suggests drought is the major cause of mortality. In some cases, fungal diseases and competition are more proximal causes (see relation between basal area and losses due to drought in Figure 5). Greater basal area reflects increases in sapwood and leaf area (Macfarlane et al., 2010; Neumann et al., 2016; O'Brien et al., 2017) that in turn require commensurate supplies of water. Greater mortality due to drought might thus be expected in stands with greater basal area, in particular when precipitation is reduced (Figure 5). It has taken five decades (since 1964) for pre‐European settlement carbon pools in aboveground biomass (assuming basal area is related to aboveground tree carbon, Burrows et al., 2002) to be restored. This is entirely in accordance with the slow‐growing nature of the dominant species (about 0.12 m^2^ ha^−1^ year^−1^ plus 0.05 m^2^ ha^−1^ year^−1^ recruitment).

Over the period for which data are available, the density of trees >40 cm diameter in managed Pilliga forests averages ~8 stems ha^−1^ (Figures 3, Figure S4)—similar to pre‐European settlement conditions (Rolls, 1999). Harvesting and pulse regeneration before 1940 increased stem density and increased coarse woody debris (Harmon et al., 1986; Woldendorp & Keenan, 2005). In the last 60 years, carbon stocks in the Pilliga have likely been similar to or even exceeded those pre‐European settlement. We note that estimates of stand structure and aboveground biomass prior to European settlement remain prone to large errors (Lunt et al., 2006).

Any future increases in frequency of large fires—exacerbated by warmer conditions and continued variability of rainfall—will challenge ability to manage for carbon and could lead to substantial loss of live tree carbon (Figure 4). Carbon stored in coarse woody debris (CWD), resulting from tree mortality and/or wildfires, and release of carbon back to the atmosphere will depend on the residence time and decomposition of CWD (Woldendorp & Keenan, 2005). Our study suggests that since 2000, fire caused a reduction in basal area from 13.3 to 7.7 m^2^ ha^−1^ in fire‐affected forests. This equates to a reduction in carbon storage of ~18 tonnes C ha^−1^ (assuming 1 m^2^ ha^−1^ equals 6.3 t ha^−1^ with 50% carbon, Burrows et al., 2002) over just two decades. The availability of consistent tree height data and reliable volume and biomass functions for Pilliga will allow testing of basal area as a suitable proxy for aboveground biomass. The global relevance of fire on *Callitris‐Eucalyptus* forests is underscored by their extent and by growing evidence of changing climates and consequences for fire frequency and intensity (Lehmann et al., 2014; Matthews et al., 2012; Russell‐Smith et al., 2013).

## 5 CONCLUSIONS

We provide evidence that small trees and recruitment are critical to the stand dynamics of semi‐arid, mixed *Callitris‐Eucalyptus* forests, including maintenance of their carbon storage. Expectations that environmental conditions will change further, with unclear effects on physiology and mortality (McDowell & Allen, 2015; Szejner et al., 2020), will likely be buffered by smaller trees. While thinning has been previously used to enhance growth of larger trees (e.g. enhanced growth pre‐1985, Figure 3), its predicted effects depend on climatic conditions and fire activity in the years to decades that follow (Horne, 1990; Whipp et al., 2012). In broad terms, re‐establishment of mixed *Callitris‐Eucalyptus* forests similar to those pre‐European settlement (Rolls, 1999) requires preventing a significant increase in fire frequency that would instead drive stands toward greater dominance by fire‐tolerant *Eucalyptus* spp. It is axiomatic that forest management must be adaptive (*sensu* Eyre et al., 2015; Lindner et al., 2014) in an era of changes in climate that can lead to changes in fire regimes. A key current discussion for management revolves around the use of thinning that would encourage development of fewer, but larger trees that are better suited as habitat and carbon stores than a much larger number of small trees. Thinning may also reduce drought stress and enhance resilience, but establishing an optimal thinning intensity for coniferous forests generally is problematic (e.g. Molina et al., 2021; Navarro‐Cerrillo et al., 2016; Sohn et al., 2016), and more for the forests studied here given a lack of rigorous experimentation. Linking the data presented here with data on weather, tree water use and soil water content would enhance knowledge of potential thinning regimes for the unique semi‐arid *Callitris* forests.

## Conflicts of interest

The authors declare no conflict of interest.

## BIOSKETCHES

**Mathias Neumann** is Assistant professor at the University of Natural Resources and Life Sciences in Vienna, interested in all aspects of trees and the fascinating ecosystems they create. A 2‐year scholarship from the Austrian Science Fund allowed him to expand his research to Australian ecosystems, focussing on semi‐arid communities. He sees international transdisciplinary research as key to highlight and tap the potential of forests and woodlands, globally.

**Mark Andrew Adams** is Professor of Bioscience and Innovation at Swinburne University of Technology. His research focus on the biology and ecology of Australia's forests, woodlands and rangelands, but he has published across fields as diverse as hydrology, soil science, biogeochemistry, atmospheric chemistry and others.

**Chris Stuart Eastaugh** is Information, Resource and Tactical Planning Project Manager at the Forestry Corporation New South Wales, responsible for management planning and field surveys in parts of the Pilliga forests.

Authors’ contributions: M.N. conceived and conducted the analysis. M.N. wrote the draft manuscript with input from C.S.E. and M.A.A. All authors revised the manuscript and gave approval for publication.

## Supporting information


Data S1

## Data Availability

The CFI data used in this analysis is available over https://figshare.com/articles/dataset/Data_used_in_Recruitment_mortality_and_growth_in_semi‐arid_conifer‐eucalypt_forest_small_trees_insure_against_fire_and_drought_/19063898. For questions please contact the corresponding author.

## References

[jbi14522-bib-0001] Ackerman, D. , Millet, D. B. , & Chen, X. (2019). Global estimates of inorganic nitrogen deposition across four decades. Global Biogeochemical Cycles, 33(1), 100–107. 10.1029/2018GB005990

[jbi14522-bib-0002] Adams, M. A. , Buckley, T. N. , Binkley, D. , Neumann, M. , & Turnbull, T. L. (2021). CO_2_, nitrogen deposition and a discontinuous climate response drive water use efficiency in global forests. Nature Communications, 12(1), 5194. 10.1038/s41467-021-25365-1 PMC8408268 34465788

[jbi14522-bib-0003] Assal, T. J. , Anderson, P. J. , & Sibold, J. (2016). Spatial and temporal trends of drought effects in a heterogeneous semi‐arid forest ecosystem. Forest Ecology and Management, 365, 137–151. 10.1016/j.foreco.2016.01.017

[jbi14522-bib-0004] Avitabile, S. C. , Callister, K. E. , Kelly, L. T. , Haslem, A. , Fraser, L. , Nimmo, D. G. , Watson, S. J. , Kenny, S. A. , Taylor, R. S. , Spence‐Bailey, L. M. , Bennett, A. F. , & Clarke, M. F. (2013). Systematic fire mapping is critical for fire ecology, planning and management: A case study in the semi‐arid Murray Mallee, south‐eastern Australia. Landscape and Urban Planning, 117, 81–91. 10.1016/j.landurbplan.2013.04.017

[jbi14522-bib-0005] Baur, G. (1990). Notes on the silviculture of major N.S.W. forest types 10. Cypress forests. Forestry Commission of New South Wales.

[jbi14522-bib-0006] Bowman, D. M. J. S. , Haverkamp, C. , Rann, K. D. , & Prior, L. D. (2018). Differential demographic filtering by surface fires: How fuel type and fuel load affect sapling mortality of an obligate seeder savanna tree. Journal of Ecology, 106(3), 1010–1022. 10.1111/1365-2745.12819

[jbi14522-bib-0007] Brodribb, T. J. , Bowman, D. M. J. S. , Grierson, P. F. , Murphy, B. P. , Nichols, S. , & Prior, L. D. (2013). Conservative water management in the widespread conifer genus Callitris. AoB PLANTS, 5, plt052. 10.1093/aobpla/plt052

[jbi14522-bib-0008] Burrows, W. H. , Henry, B. K. , Back, P. V. , Hoffmann, M. B. , Tait, L. J. , Anderson, E. R. , Menke, N. , Danaher, T. , Carter, J. O. , & McKeon, G. M. (2002). Growth and carbon stock change in eucalypt woodlands in northeast Australia: Ecological and greenhouse sink implications. Global Change Biology, 8(8), 769–784. 10.1046/j.1365-2486.2002.00515.x

[jbi14522-bib-0009] Charro, E. , Moyano, A. , & Cabezón, R. (2017). The potential of Juniperus thurifera to sequester carbon in semi‐arid forest soil in Spain. Forests, 8(9), 330. 10.3390/f8090330

[jbi14522-bib-0010] Cherlet, M. , Hutchinson, C. , Reynolds, J. , Hill, J. , Sommer, S. , & von Maltitz, G. (2018). World Atlas of Desertification. Publication Office of the European Union. 10.2760/06292

[jbi14522-bib-0011] Chidumayo, E. N. (2019). Management implications of tree growth patterns in miombo woodlands of Zambia. Forest Ecology and Management, 436(January), 105–116. 10.1016/j.foreco.2019.01.018

[jbi14522-bib-0012] Clayton‐Greene, K. , & Ashton, D. (1990). The dynamics of Callitris columellaris/Eucalyptus albens communities along the Snowy River and its tributaries in south‐eastern Australia. Australian Journal of Botany, 38(4), 403. 10.1071/BT9900403

[jbi14522-bib-0013] Cohn, J. S. , Lunt, I. D. , Ross, K. A. , & Bradstock, R. A. (2011). How do slow‐growing, fire‐sensitive conifers survive in flammable eucalypt woodlands? Journal of Vegetation Science, 22(3), 425–435. 10.1111/j.1654-1103.2011.01280.x

[jbi14522-bib-0014] Colloff, M. J. , & Baldwin, D. S. (2010). Resilience of floodplain ecosystems in a semi‐arid environment. Rangeland Journal, 32(3), 305–314. 10.1071/RJ10015

[jbi14522-bib-0015] Denham, A. J. , Vincent, B. E. , Clarke, P. J. , & Auld, T. D. (2016). Responses of tree species to a severe fire indicate major structural change to Eucalyptus–Callitris forests. Plant Ecology, 217(6), 617–629. 10.1007/s11258-016-0572-2

[jbi14522-bib-0016] Department of Planning Industry and Environment . (2020). NPWS fire history ‐ wildfires and prescribed burns. Retrieved from https://data.nsw.gov.au/data/dataset/1f694774‐49d5‐47b8‐8dd0‐77ca8376eb04

[jbi14522-bib-0017] Donohue, R. J. , McVicar, T. R. , & Roderick, M. L. (2009). Climate‐related trends in Australian vegetation cover as inferred from satellite observations, 1981‐2006. Global Change Biology, 15(4), 1025–1039. 10.1111/j.1365-2486.2008.01746.x

[jbi14522-bib-0018] Eyre, T. J. , Ferguson, D. J. , Kennedy, M. , Rowland, J. , & Maron, M. (2015). Long term thinning and logging in Australian cypress pine forest: Changes in habitat attributes and response of fauna. Biological Conservation, 186, 83–96. 10.1016/j.biocon.2015.03.009

[jbi14522-bib-0019] Farjon, A. (2005). Monograph of cupressaceae and sciadopitys. Royal Botanic Gardens.

[jbi14522-bib-0020] Forestry Commission of New South Wales . (1946). Pilliga management survey. Instructions for Forest Estimators 7/1/46. A.D. Lindsay.

[jbi14522-bib-0021] Forestry Commission of New South Wales . (n.d.). Pilliga management survey. Assessment Methods. Mr. Assessor Priestman's Methods. A.D. Lindsay.

[jbi14522-bib-0022] Grier, C. C. , Elliott, K. J. , & McCullough, D. G. (1992). Biomass distribution and productivity of Pinus edulis‐Juniperus monosperma woodlands of north‐central Arizona. Forest Ecology and Management, 50(3–4), 331–350. 10.1016/0378-1127(92)90346-b

[jbi14522-bib-0023] Guller, B. , Isik, K. , & Cetinay, S. (2012). Variations in the radial growth and wood density components in relation to cambial age in 30‐year‐old Pinus brutia Ten. at two test sites. Trees ‐ Structure and Function, 26(3), 975–986. 10.1007/s00468-011-0675-2

[jbi14522-bib-0024] Harmon, M. E. , Franklin, J. F. , Swanson, F. J. , Sollins, P. , Gregory, S. V. , Lattin, J. D. , Anderson, N. H. , Cline, S. P. , Aumen, N. G. , Sedell, J. R. , Lienkaemper, G. W. , Cromack, K. , & Cummins, K. W. (1986). Ecology of coarse woody debris in temperate ecosystems. Advances in Ecological Research, 15, 133–276. Retrieved from https://linkinghub.elsevier.com/retrieve/pii/S0065250403340024

[jbi14522-bib-0025] Hart, D. M. (1995). Litterfall and decomposition in the Pilliga State Forests, New South Wales, Australia. Australian Journal of Ecology, 20(2), 266–272. 10.1111/j.1442-9993.1995.tb00538.x

[jbi14522-bib-0026] Horne, R. (1990). Early espacement of wheatfield white cypress pine regeneration: The effect on secondary regeneration, limb size, and stand merchantability. Australian Forestry, 53(3), 160–167. 10.1080/00049158.1990.10676073

[jbi14522-bib-0027] Horne, R. , & Robinson, G. (1987). White cypress pine in N.S.W.: Growth patterns and optimal thinning regimes for 60 to 80 year old stands. Australian Forestry, 50(4), 216–223. 10.1080/00049158.1987.10676019

[jbi14522-bib-0028] Hubau, W. , Lewis, S. L. , Phillips, O. L. , Affum‐Baffoe, K. , Beeckman, H. , Cuní‐Sanchez, A. , Daniels, A. K. , Ewango, C. E. N. , Fauset, S. , Mukinzi, J. M. , Sheil, D. , Sonke, B. , Sullivan, M. J. P. , Sunderland, T. C. H. , Taedoumg, H. , Thomas, S. C. , White, T. J. T. , Abernethy, K. A. , Adu‐Bredu, S. , … Zemagho, L. (2020). Asynchronous carbon sink saturation in African and Amazonian tropical forests. Nature, 579(7797), 80–87. 10.1038/s41586-020-2035-0 32132693 PMC7617213

[jbi14522-bib-0029] Jeffrey, S. J. , Carter, J. O. , Moodie, K. B. , & Beswick, A. R. (2001). Using spatial interpolation to construct a comprehensive archive of Australian climate data. Environmental Modelling and Software, 16(4), 309–330. 10.1016/S1364-8152(01)00008-1

[jbi14522-bib-0030] Lacey, C. J. (1973). Silvicultural Characteristics of White Cypress Pine. Research Note Forestry Commission of NSW, 26, 1–51.

[jbi14522-bib-0031] Lawes, M. J. , Adie, H. , Russell‐Smith, J. , Murphy, B. , & Midgley, J. J. (2011). How do small savanna trees avoid stem mortality by fire? the roles of stem diameter, height and bark thickness. Ecosphere, 2(4), 1–13. 10.1890/ES10-00204.1

[jbi14522-bib-0032] Lehmann, C. E. R. , Anderson, T. M. , Sankaran, M. , Higgins, S. I. , Archibald, S. , Hoffmann, W. A. , Hanan, N. P. , Williams, R. J. , Fensham, R. J. , Felfili, J. , Hutley, L. B. , Ratnam, J. , San Jose, J. , Montes, R. , Russell‐Smith, J. , Ryan, C. M. , Durigan, G. , Hiernaux, P. , & Bond, W. J. (2014). Savanna vegetation‐fire‐climate relationships differ among continents. Science, 343(6170), 548–552. 10.1126/science.1247355 24482480

[jbi14522-bib-0033] Lindenmayer, D. B. , Hunter, M. L. , Burton, P. J. , & Gibbons, P. (2009). Effects of logging on fire regimes in moist forests. Conservation Letters, 2(6), 271–277. 10.1111/j.1755-263X.2009.00080.x

[jbi14522-bib-0034] Lindner, M. , Fitzgerald, J. B. , Zimmermann, N. E. , Reyer, C. , Delzon, S. , van der Maaten, E. , Schelhaas, M. J. , Lasch, P. , Eggers, J. , van der Maaten‐Theunissen, M. , Suckow, F. , Psomas, A. , Poulter, B. , & Hanewinkel, M. (2014). Climate change and European forests: What do we know, what are the uncertainties, and what are the implications for forest management? Journal of Environmental Management, 146, 69–83. 10.1016/j.jenvman.2014.07.030 25156267

[jbi14522-bib-0035] Liu, X. , He, B. , Guo, L. , Huang, L. , Yuan, W. , Chen, X. , Hao, X. , Xie, X. , Zhang, Y. , Zhong, Z. , Li, T. , & Chen, A. (2021). European carbon uptake has not benefited from vegetation greening. Geophysical Research Letters, 48(20), 1–11. 10.1029/2021GL094870

[jbi14522-bib-0036] Lopez‐Lopez, J. , Mendez Gonzalez, J. , Najera‐Luna, J. , Cerano‐Paredes, J. , Flores‐Flores, J. , & Najera‐Castro, J. (2013). Litterfall production in *Pinus halepensis* Mill. and *Pinus cembroides* Zucc. and its relatonship with some climatic factors. Agrociencia, 47(5), 497–510.

[jbi14522-bib-0037] Lunt, I. D. , Jones, N. , Spooner, P. G. , & Petrow, M. (2006). Effects of European colonization on indigenous ecosystems: Post‐settlement changes in tree stand structures in Eucalyptus‐Callitris woodlands in central New South Wales, Australia. Journal of Biogeography, 33(6), 1102–1115. 10.1111/j.1365-2699.2006.01484.x

[jbi14522-bib-0038] Macfarlane, C. , Bond, C. , White, D. A. , Grigg, A. H. , Ogden, G. N. , & Silberstein, R. (2010). Transpiration and hydraulic traits of old and regrowth eucalypt forest in southwestern Australia. Forest Ecology and Management, 260(1), 96–105. 10.1016/j.foreco.2010.04.005

[jbi14522-bib-0039] Matthews, S. , Sullivan, A. L. , Watson, P. , & Williams, R. J. (2012). Climate change, fuel and fire behaviour in a eucalypt forest. Global Change Biology, 18(10), 3212–3223. 10.1111/j.1365-2486.2012.02768.x 28741824

[jbi14522-bib-0040] McDowell, N. G. , & Allen, C. D. (2015). Darcy's law predicts widespread forest mortality under climate warming. Nature Climate Change, 5(May), 669–672. 10.1038/nclimate2641

[jbi14522-bib-0041] Middleton, N. , & Thomas, D. (1992). World Atlas of Desertification (United nations environment programme) (2nd ed.). Edward Arnold.

[jbi14522-bib-0042] Molina, A. J. , González‐Sanchis, M. , Biel, C. , & del Campo, A. D. (2021). Ecohydrological turnover in overstocked Aleppo pine plantations: Does the effect of thinning, in relation to water, persist at the mid‐term? Forest Ecology and Management, 483(November), 118781. 10.1016/j.foreco.2020.118781

[jbi14522-bib-0043] Montreal Process Implementation Group for Australia and National Forest Inventory Steering Committee . (2018). Australia's State of the Forests Report 2018. ABARES.

[jbi14522-bib-0044] Murphy, B. P. , Bradstock, R. A. , Boer, M. M. , Carter, J. , Cary, G. J. , Cochrane, M. A. , Fensham, R. J. , Russell‐Smith, J. , Williamson, G. J. , & Bowman, D. M. J. S. (2013). Fire regimes of Australia: A pyrogeographic model system. Journal of Biogeography, 40(6), 1048–1058. 10.1111/jbi.12065

[jbi14522-bib-0045] Murphy, H. T. , Bradford, M. G. , Dalongeville, A. , Ford, A. J. , & Metcalfe, D. J. (2013). No evidence for long‐term increases in biomass and stem density in the tropical rain forests of Australia. Journal of Ecology, 101(6), 1589–1597. 10.1111/1365-2745.12163

[jbi14522-bib-0046] Naudts, K. , Chen, Y. , McGrath, M. J. , Ryder, J. , Valade, A. , Otto, J. , & Luyssaert, S. (2016). Europes forest management did not mitigate climate warming. Science, 351(6273), 597–600. 10.1126/science.aad7270 26912701

[jbi14522-bib-0047] Navarro‐Cerrillo, R. M. , Sánchez‐Salguero, R. , Herrera, R. , Ruiz, C. J. C. , Moreno‐Rojas, J. M. , Manzanedo, R. D. , & López‐Quintanilla, J. (2016). Contrasting growth and water use efficiency after thinning in mixed Abies pinsapo‐Pinus pinaster‐Pinus sylvestris forests. Journal of Forest Science, 62(2), 53–64. 10.17221/104/2015-JFS

[jbi14522-bib-0048] Neumann, M. , Moreno, A. , Mues, V. , Härkönen, S. , Mura, M. , Bouriaud, O. , Lang, M. , Achten, W. M. J. , Thivolle‐Cazat, A. , Bronisz, K. , Merganic, J. , Decuyper, M. , Alberdi, I. , Astrup, R. , Mohren, F. , & Hasenauer, H. (2016). Comparison of carbon estimation methods for European forests. Forest Ecology and Management, 361, 397–420. 10.1016/j.foreco.2015.11.016

[jbi14522-bib-0049] Ngugi, M. R. , Botkin, D. B. , Doley, D. , Cant, M. , & Kelley, J. (2013). Restoration and management of callitris forest ecosystems in Eastern Australia: Simulation of attributes of growth dynamics, growth increment and biomass accumulation. Ecological Modelling, 263, 152–161. 10.1016/j.ecolmodel.2013.05.004

[jbi14522-bib-0050] Ngugi, M. R. , Doley, D. , Cant, M. , & Botkin, D. B. (2015). Growth rates of Eucalyptus and other Australian native tree species derived from seven decades of growth monitoring. Journal of Forestry Research, 26(4), 811–826. 10.1007/s11676-015-0095-z

[jbi14522-bib-0051] Nguyen, T. T. , & Baker, P. J. (2016). Structure and composition of deciduous dipterocarp forest in Central Vietnam: Patterns of species dominance and regeneration failure. Plant Ecology and Diversity, 9(5–6), 589–601. 10.1080/17550874.2016.1210261

[jbi14522-bib-0052] Nolan, R. H. , Sinclair, J. , Waters, C. M. , Mitchell, P. J. , Eldridge, D. J. , Paul, K. I. , Roxburgh, S. , Butler, D. W. , & Ramp, D. (2019). Risks to carbon dynamics in semi‐arid woodlands of eastern Australia under current and future climates. Journal of Environmental Management, 235(May 2018), 500–510. 10.1016/j.jenvman.2019.01.076 30711835

[jbi14522-bib-0053] O'Brien, M. J. , Engelbrecht, B. M. J. , Joswig, J. , Pereyra, G. , Schuldt, B. , Jansen, S. , Kattge, J. , Landhä usser, S. M. , Levick, S. R. , Preisler, Y. , Väänänen, P. , & Macinnis‐Ng, C. (2017). A synthesis of tree functional traits related to drought‐induced mortality in forests across climatic zones. Journal of Applied Ecology, 54(6), 1669–1686. 10.1111/1365-2664.12874

[jbi14522-bib-0054] Paloschi, R. A. , Ramos, D. M. , Ventura, D. J. , Souza, R. , Souza, E. , Morellato, L. P. C. , Nobrega, R. L. B. , Coutinho, I. A. C. , Verhoef, A. , Körting, T. S. , & Borma, L. D. S. (2021). Environmental drivers of water use for caatinga woody plant species: Combining remote sensing phenology and sap flow measurements. Remote Sensing, 13(1), 1–18. 10.3390/rs13010075 36817948

[jbi14522-bib-0055] Prior, L. D. , & Bowman, D. M. J. S. (2014). Big eucalypts grow more slowly in a warm climate: Evidence of an interaction between tree size and temperature. Global Change Biology, 20(9), 2793–2799. 10.1111/gcb.12540 24469908

[jbi14522-bib-0056] Prior, L. D. , Grierson, P. F. , McCaw, W. L. , Tng, D. Y. P. , Nichols, S. C. , & Bowman, D. M. J. S. (2012). Variation in stem radial growth of the Australian conifer, Callitris columellaris, across the world's driest and least fertile vegetated continent. Trees ‐ Structure and Function, 26(4), 1169–1179. 10.1007/s00468-012-0693-8

[jbi14522-bib-0057] R Development Core Team . (2021). R: A language and environment for statistical computing. R Foundation for Statistical Computing. Retrieved from http://www.r‐project.org/

[jbi14522-bib-0058] Rau, B. M. , Johnson, D. W. , Blank, R. R. , & Chambers, J. C. (2009). Soil carbon and nitrogen in a Great Basin pinyon‐juniper woodland: Influence of vegetation, burning, and time. Journal of Arid Environments, 73(4–5), 472–479. 10.1016/j.jaridenv.2008.12.013

[jbi14522-bib-0059] Rolls, E. C. (1999). Land of grass: The loss of Australia's grasslands. Australian Geographical Studies, 37(3), 197–213. 10.1111/1467-8470.00079

[jbi14522-bib-0060] Ross, K. A. , Lunt, I. D. , Bradstock, R. A. , Bedward, M. , & Ellis, M. V. (2012). Did historical tree removal promote woody plant encroachment in Australian woodlands? Journal of Vegetation Science, 23(2), 304–312. 10.1111/j.1654-1103.2011.01356.x

[jbi14522-bib-0061] Russell‐Smith, J. , Cook, G. D. , Cooke, P. M. , Edwards, A. C. , Lendrum, M. , Meyer, C. P. , & Whitehead, P. J. (2013). Managing fire regimes in north Australian savannas: Applying Aboriginal approaches to contemporary global problems. Frontiers in Ecology and the Environment, 11(Suppl. 1), e55–e63. 10.1890/120251

[jbi14522-bib-0062] Ryan, C. M. , Williams, M. , & Grace, J. (2011). Above‐ and belowground carbon stocks in a miombo woodland landscape of mozambique. Biotropica, 43(4), 423–432. 10.1111/j.1744-7429.2010.00713.x

[jbi14522-bib-0063] Sohn, J. A. , Saha, S. , & Bauhus, J. (2016). Potential of forest thinning to mitigate drought stress: A meta‐analysis. Forest Ecology and Management, 380, 261–273. 10.1016/j.foreco.2016.07.046

[jbi14522-bib-0064] Stephenson, N. L. , Das, A. J. , Condit, R. , Russo, S. E. , Baker, P. J. , Beckman, N. G. , Coomes, D. A. , Lines, E. R. , Morris, W. K. , Ruger, N. , Alvarez, E. , Blundo, C. , Bunyavejchewin, S. , Chuyong, G. , Davies, S. J. , Duque, A. , Ewango, C. N. , & Zavala, M. A. (2014). Rate of tree carbon accumulation increases continuously with tree size. Nature, 507(7490), 90–93. 10.1038/nature12914 24429523

[jbi14522-bib-0065] Szejner, P. , Belmecheri, S. , Ehleringer, J. R. , & Monson, R. K. (2020). Recent increases in drought frequency cause observed multi‐year drought legacies in the tree rings of semi‐arid forests. Oecologia, 192(1), 241–259. 10.1007/s00442-019-04550-6 31686228

[jbi14522-bib-0066] Thompson, W. A. , & Eldridge, D. J. (2005). White cypress pine (Callitris glaucophylla): A review of its roles in landscape and ecological processes in eastern Australia. Australian Journal of Botany, 53(6), 555. 10.1071/BT04115

[jbi14522-bib-0067] Trauernicht, C. , Murphy, B. P. , Portner, T. E. , & Bowman, D. M. J. S. (2012). Tree cover‐fire interactions promote the persistence of a fire‐sensitive conifer in a highly flammable savanna. Journal of Ecology, 100(4), 958–968. 10.1111/j.1365-2745.2012.01970.x

[jbi14522-bib-0068] Trauernicht, C. , Murphy, B. P. , Prior, L. D. , Lawes, M. J. , & Bowman, D. M. J. S. (2016). Human‐imposed, fine‐grained patch burning explains the population stability of a fire‐sensitive conifer in a frequently burnt Northern Australia Savanna. Ecosystems, 19(5), 896–909. 10.1007/s10021-016-9973-2

[jbi14522-bib-0069] Trumper, K. , Ravilious, C. , & Dickson, B. (2008). Carbon in drylands: desertification, climate change and carbon finance. A UNEP‐UNDP‐UNCCD Technical Note for Discussions at CRIC, 7, 1–12.

[jbi14522-bib-0070] Turner, B. J. (1968). Sampling with variable radius plots. In Paper Presented to the British Commonwealth Forestry Conference. Forestry Commission of N.S.W.

[jbi14522-bib-0071] Turner, J. C. , Friedel, M. H. , & Neumann, M. (2021). Phyllode fall and nutrient content in a mulga (*Acacia aneura* F.Muell. ex Benth.) community in central Australia in response to rainfall. The Rangeland Journal, 43(1), 1. 10.1071/RJ21007

[jbi14522-bib-0072] Van Meerbeek, K. , Jucker, T. , & Svenning, J. C. (2021). Unifying the concepts of stability and resilience in ecology. Journal of Ecology, 109(9), 3114–3132. 10.1111/1365-2745.13651

[jbi14522-bib-0073] Werneck, F. P. , Nogueira, C. , Colli, G. R. , Sites, J. W. , & Costa, G. C. (2012). Climatic stability in the Brazilian Cerrado: Implications for biogeographical connections of South American savannas, species richness and conservation in a biodiversity hotspot. Journal of Biogeography, 39(9), 1695–1706. 10.1111/j.1365-2699.2012.02715.x

[jbi14522-bib-0074] Whipp, R. (2009). Historical vegetation change in relation to forest management in the Pilliga State Forests of northern NSW. Charles Sturt University.

[jbi14522-bib-0075] Whipp, R. K. , Lunt, I. D. , Spooner, P. G. , & Bradstock, R. A. (2012). Changes in forest structure over 60 years: Tree densities continue to increase in the Pilliga forests, New South Wales, Australia. Australian Journal of Botany, 60(1), 1–8. 10.1071/BT11191

[jbi14522-bib-0076] Woldendorp, G. , & Keenan, R. J. (2005). Coarse woody debris in Australian forest ecosystems: A review. Austral Ecology, 30(8), 834–843. 10.1111/j.1442-9993.2005.01526.x

[jbi14522-bib-0077] Yan, K. , Park, T. , Yan, G. , Chen, C. , Yang, B. , Liu, Z. , Nemani, R. , Knyazikhin, Y. , & Myneni, R. (2016). Evaluation of MODIS LAI/FPAR Product Collection 6. Part 1: Consistency and Improvements. Remote Sensing, 8(5), 359. 10.3390/rs8050359

