# Supplementary material for: Recruitment, mortality and growth in semi‐arid conifer‐eucalypt forest: Small trees insure against fire and drought
Source: J Biogeogr. 2022 Dec 13;50(2):291–301. doi: 10.1111/jbi.14522 (PMC10107837; doi:10.1111/jbi.14522)
Supplement: Supplementary file 1 — Data S1 [file JBI-50-291-s001.docx]

Supplement

**Recruitment, mortality and growth in a semi-arid conifer-eucalypt forest – small trees are insurance against fire and drought**

Mathias Neumann ^1,2^*, Chris S. Eastaugh ^3^, Mark A. Adams ^1^

^1^ Faculty of Science, Engineering and Technology, Swinburne University of Technology, Melbourne, Australia

^2^ Institute of Silviculture, University of Natural Resources and Life Sciences, 1190 Vienna, Austria

^3^ Forestry Corporation New South Wales, Dubbo, Australia

* Corresponding author: mathias.neumann@boku.ac.at

Table S1: Stand structure based on CFI measurements and other information (see main text). Median and 50% interquartile range are shown for basal area and stem density. Losses of stem density are separated by cause based on data for 1964-2019. Trees < 10 cm DBH were only assessed three times.

| Year | Basal area | Stem density | Losses of stem density (ha^-1^ year^-1^) | | | | | Recruitment | Stem density |
| --- | --- | --- | --- | --- | --- | --- | --- | --- | --- |
|  | (m^2^ ha^-1^) | (ha^-1^) | Harvest | Fire | Wind | Drought | All | (ha^-1^ year^-1^) | <10 cm (ha^-1^) |
| pre-settlement | 12.0 ± 1.1 | 44 ± 6 | - | - | - | - | - | - | - |
| 1940s | 4.2 | 188 | - | - | - | - | - | - | - |
| 1964 | 12.0 ± 2.0 | 344 ± 145 | - | - | - | - | - | - | - |
| 1972/73 | 12.1 ± 2.2 | 338 ± 145 | 1.92 | 0.11 | 0.28 | 1.91 | 4.23 | 3.67 | 1520 ± 1245 |
| 1984/85 | 13.3 ± 2.9 | 368 ± 145 | 1.06 | 0.07 | 0.23 | 0.92 | 2.29 | 4.95 | 1680 ± 1011 |
| 2000/01 | 15.1 ± 3.2 | 426 ± 135 | 0.73 | 0.36 | 0.17 | 1.47 | 2.72 | 6.49 | - |
| 2019 | 13.8 ± 3.2 | 398 ± 110 | 0.85 | 0.97 | 0.11 | 4.30 | 6.23 | 4.48 | 550 ± 340 |

Table S2: Contributions of recruitment and mortality to net growth (m^2^ ha^-1^ year^-1^). Values are medians based on CFI plots. All is sum of losses to harvesting, fire, wind and other causes. ‘Net’ is the balance of increment + recruitment – all losses.

| Period | Harvest | Fire | Wind | Drought | All | Increment | Recruitment | Net |
| --- | --- | --- | --- | --- | --- | --- | --- | --- |
| 1964-73 | 0.096 | 0.002 | 0.014 | 0.049 | 0.162 | 0.125 | 0.035 | -0.002 |
| 1972-85 | 0.055 | 0.002 | 0.007 | 0.031 | 0.094 | 0.143 | 0.057 | 0.105 |
| 1984-01 | 0.032 | 0.009 | 0.005 | 0.041 | 0.087 | 0.110 | 0.074 | 0.097 |
| 2000-19 | 0.036 | 0.033 | 0.004 | 0.132 | 0.206 | 0.088 | 0.047 | -0.070 |

Table S3: Summary of measurements for trees 5-10 cm DBH. Complete tallies made in the 1980s allow calculating stem density and basal area and the surviving trees were assessed again in 2019 to derive increment and survival. We show the species proportion at stem density.

| Number plots | Measured trees | Time period | Stem density 5-10 cm (ha^-1^) | Basal area (m^2^ ha^-1^) | Diameter increment (cm year^-1^) | | Survival rate (%) | |  |
| --- | --- | --- | --- | --- | --- | --- | --- | --- | --- |
| 29 | 889 | 1984-2019 | 409 ± 367 | 1.73 ± 1.50 | 0.109 ± 0.127 | | 55.1 | |  |
| Share in stem density (%) | |  |  |  |  | | |  | |
| *Callitris glaucophylla* | *Callitris endlicherii* | *Eucalyptus crebra* | *Allocasuarina luehmannii* | *Eucalyptus pilligaensis* | *Eucalyptus blakelyi* | other | |  |  |
| 48.8 | 3.7 | 4.2 | 35.1 | 0.8 | 0.6 | 6.9 | |  |  |

Table S4: Prevailing climatic conditions for the four measurement periods. For each measurement period, average annual precipitation (P), average daily temperatures (T, calculated as mean of daily maximum and minimum temperature) and Potential Evapotranspiration (PET, using the Penman-Monteith approach) are shown, along with anomalies (Abatzoglou, Dobrowski, Parks, & Hegewisch, 2018; Jeffrey, Carter, Moodie, & Beswick, 2001). We calculated Aridity Index (AI) as the ratio of P and PET. We extracted annual values using the PSP locations and calculated averages per period. The number of fires per year (1982/83, 2006/07 and 2017/18) affecting the PSPs are also shown.

| Period | P (mm) | T (˚C) | PET (mm) | AI (mm/mm) | P anomaly (mm) | T anomaly (˚C) | Fires per year |
| --- | --- | --- | --- | --- | --- | --- | --- |
| 1964-72 | 547 | 17.63 | 1555 | 0.35 | -13.07 | -0.76 | 0.00 |
| 1973-84 | 596 | 18.60 | 1538 | 0.39 | 36.57 | 0.21 | 0.08 |
| 1985-00 | 589 | 18.44 | 1571 | 0.37 | 29.17 | 0.05 | 0.00 |
| 2001-19 | 516 | 18.63 | 1704 | 0.30 | -43.77 | 0.24 | 0.11 |
| 1964-19 | 560 | 18.39 | 1600 | 0.35 |  |  |  |

Table S5: Mixed models of basal area increment, recruitment and losses using basal area, BA (m^2^ ha^-1^), quadratic mean tree diameter, DG (cm) and annual average precipitation, P (mm) as fixed effects and observation period as random effects. We calculated separate models for losses due to harvesting, fire, wind and drought. We show coefficient of determination, R^2^, for fixed and random effects and *P* value of the overall regression. Bold covariates are significant (*P* < 0.05).

| Variable | Equation | *P* value | Fixed R^2^ | Random R^2^ |
| --- | --- | --- | --- | --- |
| Increment | -0.0266 **+ 0.00248 BA - 0.00370 DG + 0.000347 P** | < 0.001 | 0.184 | 0.062 |
| Recruitment | -0.00905 **- 0.00160 BA - 0.00968 DG + 0.000163 P** | < 0.001 | 0.059 | 0.051 |
| Losses | -0.138 **+ 0.00928 BA - 0.00239 DG** + 0.000370 P | < 0.001 | 0.075 | 0.103 |
| Harvest | 0.0793 + 0.00215 BA - 0.00114 DG - 8.05 10^-5^ P | 0.111 | 0.011 | 0.004 |
| Fire | 0.0493 + 0.000383 BA - 0.000298 DG - 5.22 10^-5^ P | 0.930 | 0.001 | 0.062 |
| Wind | -0.00333 + 0.000395 BA - 0.000143 DG + 1.64 10^-5^ P | 0.357 | 0.006 | 0.000 |
| Drought | **-0.182 + 0.0106 BA - 0.00212 DG + 0.000326 P** | < 0.001 | 0.155 | 0.262 |


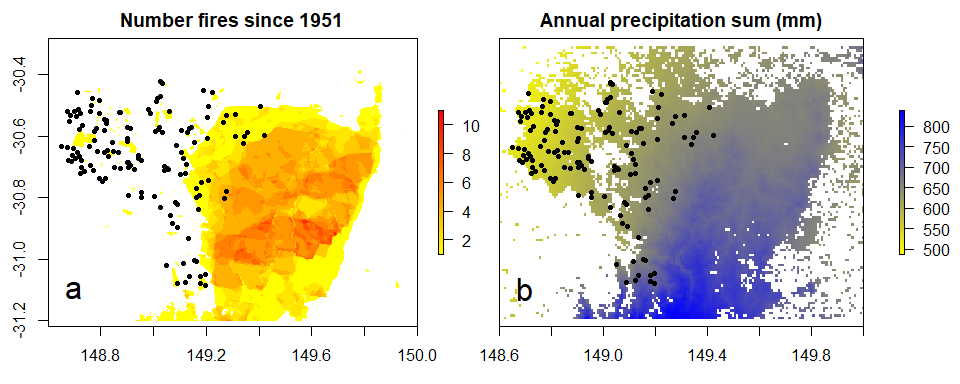


Fig. S1: (a) Number of fires (wildfires and prescribed burns) since 1951 (Department of Planning Industry and Environment, 2020) and (b) annual precipitation 1970-2000 (Jeffrey et al., 2001). In (b) non-forest areas are masked (Friedl et al., 2010). PSP locations are indicated by dots. Map projection is WGS84 (EPSG:4326).


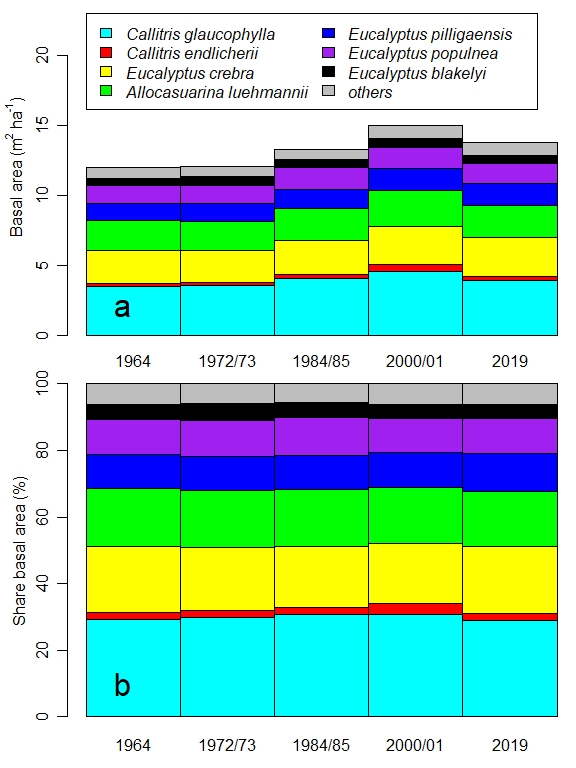


Fig. S2: Species composition of basal area (a) and the relative share (b) over the five measurement periods.


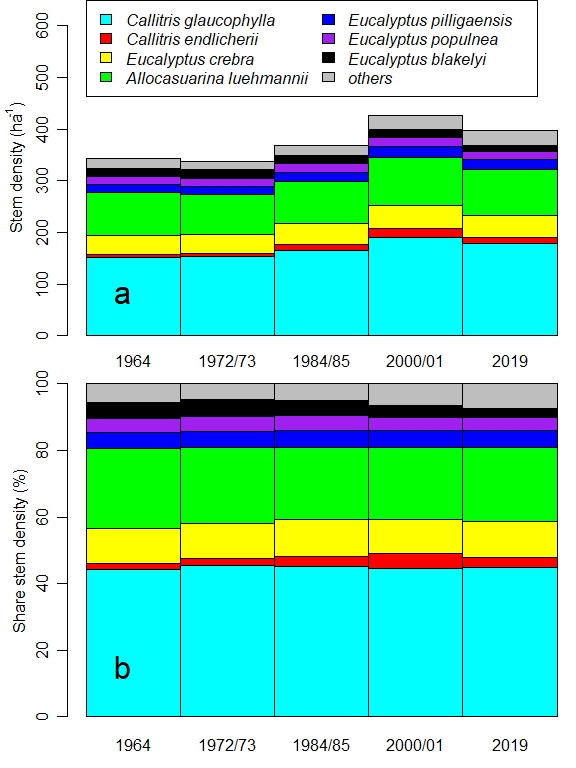


Fig. S3: Species composition of stem density (a) and the relative share (b) over five measurement periods.


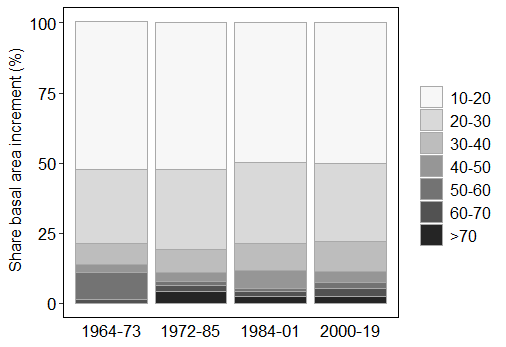


Fig. S4: Proportional contributions of diameter classes to basal area increments.


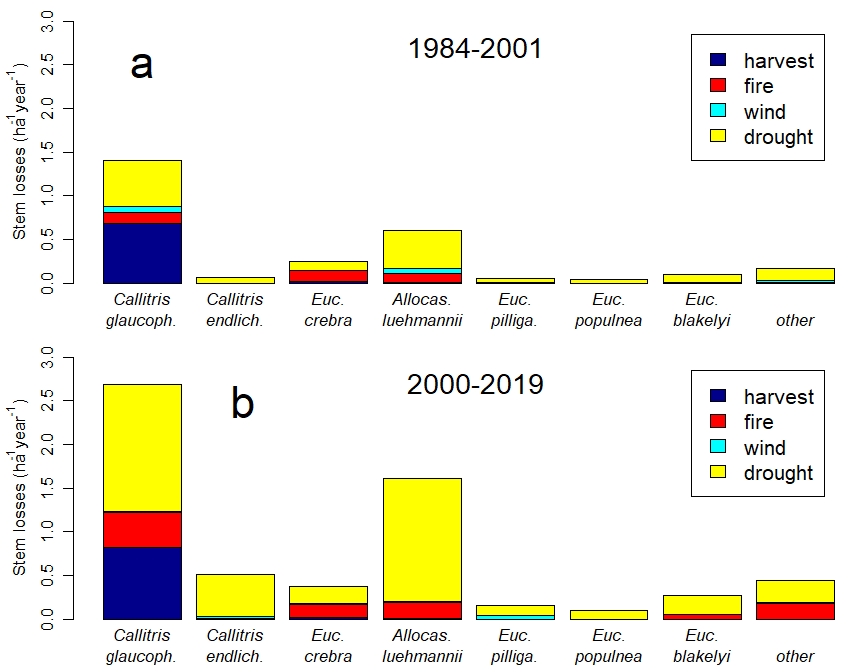


Fig. S5: Contributions of different causes of mortality to overall losses of stem density by tree species (for full names see figure S2). We show period 1984-2001 in (a) and 2000-19 in (b).


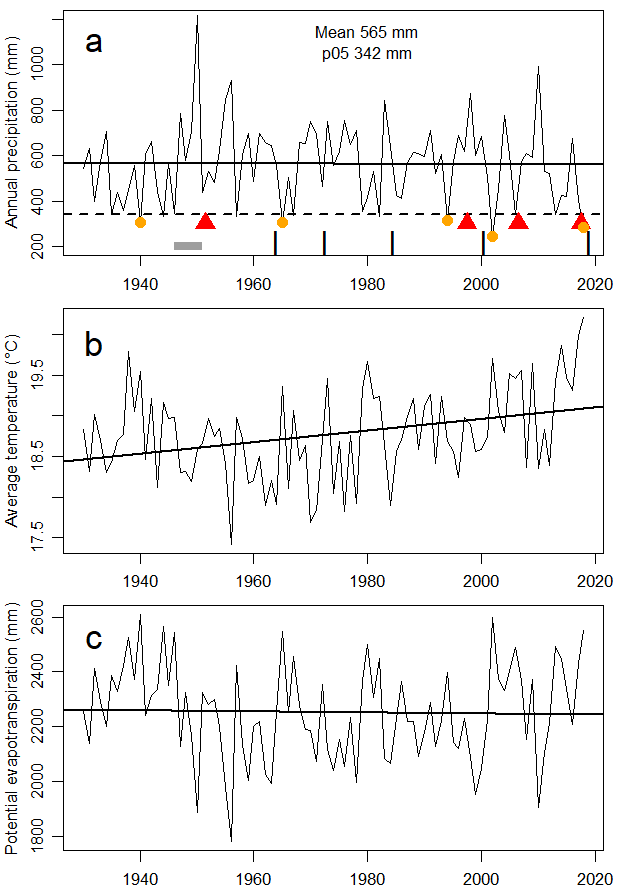


Fig. S6: Relationship of fire history to annual precipitation (a) from 1930 until 2019 (Jeffrey et al., 2001). Red triangles indicate major wildfires. Orange dots indicate years with precipitation less than 5% quantile (dashed line). Solid line is the linear trend in annual precipitation and the dashed line the 5^th^ percentile. Vertical black bars indicate CFI measurements. For context we show Lindsay’s 1940s survey (grey bar). We also show average temperazure (b) and potential evapotranspiration sum (c) to evaluate change in aridity index (precipiation / potential evapotranspiration).


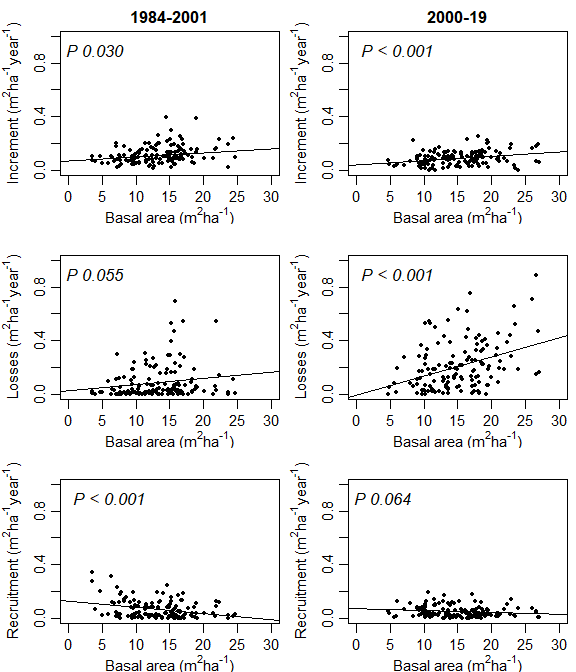


Fig. S7: Relationships of basal area to basal area increment (top), losses due to mortality and harvesting (middle) and recruitment (bottom) for two time periods, 1984-2001 (left) and 2000-2019 (right). Number of observations is 136 for each period. Linear trend lines and their *P* values are shown for reference.

**References**

Abatzoglou, J. T., Dobrowski, S. Z., Parks, S. A., & Hegewisch, K. C. (2018). TerraClimate, a high-resolution global dataset of monthly climate and climatic water balance from 1958-2015. *Scientific Data*, *5*, 1–12. https://doi.org/10.1038/sdata.2017.191

Department of Planning Industry and Environment. (2020). NPWS fire history - wildfires and prescribed burns. Retrieved from https://data.nsw.gov.au/data/dataset/1f694774-49d5-47b8-8dd0-77ca8376eb04

Friedl, M., Sulla-Menashe, D., Tan, B., Schneider, A., Ramankutty, N., Sibley, A., & Huang, X. (2010). MODIS Collection 5 global land cover: Algorithm refinements and characterization of new datasets. *Remote Sensing of Environment*, *114*(1), 168–182. https://doi.org/10.1016/j.rse.2009.08.016

Jeffrey, S. J., Carter, J. O., Moodie, K. B., & Beswick, A. R. (2001). Using spatial interpolation to construct a comprehensive archive of Australian climate data. *Environmental Modelling and Software*, *16*(4), 309–330. https://doi.org/10.1016/S1364-8152(01)00008-1
